# Enhanced *in vitro* biocompatibility and osteogenesis of titanium substrates immobilized with dopamine-assisted superparamagnetic Fe_3_O_4_ nanoparticles for hBMSCs

**DOI:** 10.1098/rsos.172033

**Published:** 2018-08-01

**Authors:** Zhenfei Huang, Zhihong Wu, Bupeng Ma, Lingjia Yu, Yu He, Derong Xu, Yuanhao Wu, Hai Wang, Guixing Qiu

**Affiliations:** 1 Department of Orthopaedics, Peking Union Medical College Hospital, Chinese Academy of Medical Sciences and Peking Union Medical College, Beijing 100730, People's Republic of China; 2 Central Laboratory, Peking Union Medical College Hospital, Chinese Academy of Medical Sciences and Peking Union Medical College, Beijing 100730, People's Republic of China

**Keywords:** osteogenesis, superparamagnetic Fe_3_O_4_ nanoparticles, dopamine-assisted coating

## Abstract

Titanium (Ti) is an ideal bone substitute due to its superior bio-compatibility and remarkable corrosion resistance. However, in order to improve the osteoconduction and osteoinduction capacities in clinical applications, different kinds of surface modifications are typically applied to Ti alloys. In this study, we fabricated a tightly attached polydopamine-assisted Fe_3_O_4_ nanoparticle coating on Ti with magnetic properties, aiming to improve the osteogenesis of the Ti substrates. The PDA-assisted Fe_3_O_4_ nanoparticle coatings were characterized by scanning electron microscopy, energy dispersive spectroscopy, atomic force microscopy and water contact angle measurements. The cell attachment and proliferation rate of the human bone mesenchymal stem cells (hBMSCs) on the Ti surface significantly improved with the Fe_3_O_4_/PDA coating when compared with the pure Ti without a coating. Furthermore, the results of *in vitro* alkaline phosphatase (ALP) activity at 7 and 14 days and alizarin red S staining at 14 days showed that the Fe_3_O_4_/PDA coating on Ti promoted the osteogenic differentiation of hBMSCs. Moreover, hBMSCs co-cultured with the Fe_3_O_4_/PDA-coated Ti for approximately 14 days also exhibited a significantly higher mRNA expression level of ALP, osteocalcin and runt-related transcription factor-2 (RUNX2). Our *in vitro* results revealed that the present PDA-assisted Fe_3_O_4_ nanoparticle surface coating is an innovative method for Ti surface modification and shows great potential for clinical applications.

## 1. Introduction

The superior biocompatibility and corrosion resistance of titanium (Ti) made it one of the most promising materials for load-bearing and bone-contacting medical substitution in clinical practice [1]. Ti implants can be placed into defect sites to treat bone defects caused by trauma or tumours. The good interfacial interaction of the bone–implant interface is a critical factor for biomechanical stability [2]. However, there is an insufficient interaction between the Ti implant surface and the surrounding tissues and bone cells, which needs to be improved. Thus, the development of appropriate surface modification techniques for Ti implants with high osteoconduction and osteoinduction has attracted great attention for bone regeneration [2–6].

Although varieties of surface modification for Ti have been investigated as alternatives to improve osteoconduction and osteoinduction [2–8], there is still ample space for improvement to meet urgent clinical requirements. Recently, magnetism has been found to positively impact cellular responses [9–11]. Plenty of magnetic composite materials that integrate with magnetic nanoparticles (MNPs) have been manufactured to accelerate the repair and regeneration of engineering tissues [10,12–17]. For instance, calcium phosphate cements macerated with ultrafine MNPs have significant promotional effects on cell adhesion, proliferation and differentiation [15]. Additionally, biopolymer nanocomposite scaffolds mixed with MNPs increased the adhesion and differentiation of osteoblastic cells *in vitro* and bone formation *in vivo* [9,12,14]. From the above examples, MNPs can be considered a valid stimulus to bone fracture healing and new bone formation.

Physical deposition techniques, including thermal plasma spraying [18], pulsed laser deposition [19], ion-beam-assisted deposition [20] and electrophoretic deposition [21] have been widely adopted for coating Ti implants for orthopaedic applications. Nevertheless, most of the physical deposition techniques require a complex chemical synthesis process or expensive instruments and apparatuses [2]. Worse, the high temperature of plasma spraying may damage the magnetic properties of the MNPs. Therefore, in recent years, a facile and versatile surface modification technique based on the mussel-inspired polymerization of dopamine (PDA) at alkaline pH on various substrates has attracted considerable attention and has been applied to the surface modification of Ti implants for orthopaedic applications [22–24]. Additionally, many nanoparticles including MNPs can be formed on the PDA shell *in situ*, which promotes tight bonding between the nanoparticle shell and the Ti substrate [2,24]. Kang *et al*. have reported a one-step procedure for surface modification [25]. This process can be applied to the surface of diverse materials without further time-consuming chemical syntheses. Zheng and co-workers successfully synthesized a polydopamine (PDA) coating on the surface of magnetic Fe_3_O_4_ particles, which are highly biocompatible and have a photothermal effect [24].

**Scheme 1.**
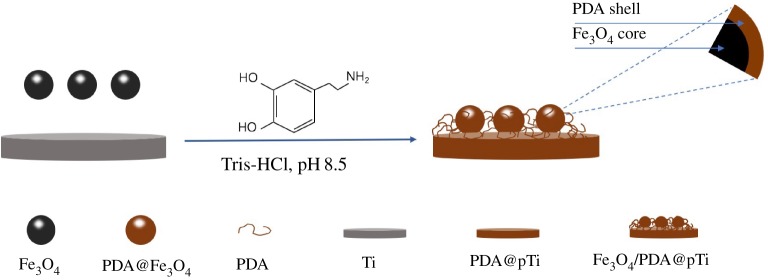
Schematic illustration of PDA-assisted immobilization of Fe_3_O_4_ nanoparticles on surfaces of titanium substrates.

In this work, we chose dopamine as a binding agent on the Ti substrate to build a bridge between the magnetic Fe_3_O_4_ particles and the metal matrix. Interestingly, our results revealed enhanced cell adhesion, proliferation and differentiation of the human bone mesenchymal stem cells (hBMSCs) when compared to a pure PDA-coated Ti substrate at an equivalent condition. To the best of our knowledge, the enhanced osteogenic effect of PDA-assisted Fe_3_O_4_ particles coated onto the Ti substrate (PDA/Fe_3_O_4_@pTi) has never been demonstrated. *In vitro* assays were performed to determine whether the PDA/Fe_3_O_4_ coating could endow Ti implants with both good osteoconductive and osteoinductive abilities. The process is schematically depicted in scheme 1.

## 2. Material and methods

### 2.1. Chemicals

3,4-Dihydroxyphenethylamine hydrochloride (DOPA) and tris (hydroxymethyl) aminomethane (Tris) were purchased from Aladdin Industrial Corporation; poly(4-styrenesulfonic acid-co-maleic acid, SS : MA = 1 : 1) sodium salt (PSSMA 1 : 1), ferric chloride hexahydrate (FeCl_3_•6H_2_O), ethylene glycol (EG), and anhydrous sodium acetate were purchased from Sigma-Aldrich. All chemicals were of analytical grade and used without further purification. All solutions were prepared using deionized (DI) water, which was obtained from a Millipore Elix pure water system. Ti samples were prepared by cutting cylindrical rods (15 mm in diameter and 10 cm in length) of pure Ti (Shanghai Baosteel Co. Ltd, China) into a disc-like shape with 2 mm thickness with a discharge cutting device. All samples were ultrasonically cleaned sequentially in 1 M HCl, acetone, ethyl alcohol and DI water for approximately 15 min before coating with dopamine.

### 2.2. Preparation of Fe_3_O_4_ nanoparticles

The Fe_3_O_4_ nanoparticles were synthesized by a modified one-step solvothermal method as previously described [26]. Briefly, 0.5 g of PSSMA (1 : 1) was dissolved in EG (20 ml), and then, the FeCl_3_•6H_2_O (0.54 g) and sodium acetate (1.5 g) were added into this solution. The obtained homogeneous red brown solution was then sealed in a Teflon-lined stainless-steel autoclave and heated at 200°C for 12 h. After the reaction, the dark products obtained were separated from the solutions by a magnet, washed with DI water at least three times, and then dried in vacuum for 24 h.

### 2.3. Preparation of Fe_3_O_4_/PDA@pTi

The coating of Fe_3_O_4_/PDA onto titanium substrates (pTi) was synthesized by a one-step method at room temperature [25]. The Fe_3_O_4_ nanoparticles (20 mg) and 3,4-dihydroxyphenethylamine hydrochloride (dopamine hydrochloride) were dispersed in the Tris buffer solution (10 mM, pH 8.5, 100 ml) to 2 mg ml^−1^. Additionally, the PDA-Tris buffer solution (10 mM, pH 8.5, 100 ml, 2 mg ml^−1^) was prepared for the control experiment. Then, the pTi samples were immersed into the solutions for 24 h at room temperature in a thermostatic shaker at 150 r.p.m. After immersion, the final products were ultrasonically treated in DI water and dried by nitrogen gas to obtain the desired Fe_3_O_4_/PDA-coated pTi (Fe_3_O_4_/PDA@pTi) and PDA-coated pTi (PDA@pTi), respectively. To tailor the thickness and stability of the Fe_3_O_4_/PDA, we altered the time of reaction to 24 h while the mass of Fe_3_O_4_ nanoparticles was fixed to 20 mg according to the pre-experiment.

### 2.4. Surface characterization

Surface morphology was observed using a scanning electron microscope (SEM, Quanta FEG 250) at an accelerating voltage of 10 kV. The chemical compositions of the samples were identified by energy dispersive spectroscopy (EDS, Oxford, IE250X-Max50, UK). The distribution of chemical elements on the surface of the modified substrates was monitored by EDS elemental mapping. The surface roughness was observed using an atomic force microscope in tapping mode (atomic force microscopy (AFM), Agilent-5100, USA). The water contact angle was measured by using a contact angle goniometer (Dataphysics, Germany) [27]. Briefly, 5 µl of DI water was dropped onto the sample surfaces. Optical images of the water droplet were taken after 1 min, and the ascending angles were measured. The magnetic properties of the scaffolds were measured by a vibrating sample magnetometer (VSM, Lake Shore VSM-7307) in an applied magnetic field of ±20 kOe at room temperature, in terms of saturation magnetization and hysteresis loops [15]. The VSM was calibrated using a high purity nickel sphere standard supplied by the company. The mechanical stability of Fe_3_O_4_/PDA coating was evaluated using an ultrasonication test (40 kHz) as previously described [27]. Briefly, before and after an hour of ultrasonication, the EDS was used to identify the content of the Fe element at the same spot on the samples.

### 2.5. Cell culture and cell seeding

Human bone marrow mesenchymal stem cells (hBMSCs) were purchased from 307-Ivy Translational Medicine Center (Beijing, China) and cultured in Basal medium for hBMSCs (Cyagen, HUXMA-90011, USA) supplemented with 10% fetal bovine serum (FCS; Invitrogen, Carlsbad, CA, USA), 1% penicillin/streptomycin and glutamine (Cyagen, USA) in an incubator (Sanyo, Japan) with 5% CO_2_ at 37°C and saturated humidity. After growing to 80% confluence, hBMSCs were digested by 0.25% trypsin (Solarbio Science & Technology Co., Ltd, Beijing) for further use. The culture medium was exchanged every 2–3 days. The cells at passages 3–5 were used for proliferation and differentiation experiments.

### 2.6. Cytotoxicity and proliferation

The cytotoxicity of the samples was assayed by the LIVE/DEAD kit for mammalian cells (Invitrogen). The kit contained two probes: calcein AM and ethidium homodimer (EthD-1). Calcein AM was converted to calcein by active intracellular esterase of living cells to generate green fluorescence. EthD-1 passed through broken cell membranes and produced red fluorescence. After the cells were seeded and incubated for 1, 3 and 7 days, the samples were taken from the 24-well plates and rinsed three times with PBS. Then, 500 µl of the working solution was immediately added to each sample and incubated in darkness at 37°C for 20 min. Subsequently, all samples were observed with a confocal laser scanning microscope (Leica, TCS, SP5, Germany). The cell area and cell density were calculated with six different region of interest (ROI) fields of each sample (*n* = 3) from immunofluorescence imaging using ImageJ software (NIH, Bethesda, MD, USA).

Cell proliferation was measured by a cell count kit-8 (CCK-8) assay. Briefly, 1, 3 and 7 days after cell seeding, the samples were transferred to new 24-well plates and rinsed with PBS gently to remove loosely attached cells, followed by adding 500 µl of culture medium to each well. Then, 50 µl of CCK-8 (Dojindo Molecular Technologies Inc., Minato-ku, Tokyo, Japan) solution was added into each well and incubated at 37°C for 30 min. Subsequently, 100 µl of the reaction solution was transferred into a new 96-well plate, and the optical density was measured at 450 nm by a microplate reader.

### 2.7. Cell morphology

For fluorescence observation, the cells were rinsed three times with PBS and stained by rhodamine-phalloidin (Cytoskeleton, Inc.) for 30 min at RT for visualization of filamentous actin (F-actin) after incubation for 24 h. Then, the nuclei were counterstained with 4′,6 diamidino-2-phenylindole (DAPI). Images were captured using a confocal laser scanning microscope (Leica, TCS, SP5, Germany). The cell overall area to the nucleus area (CN ratio) was calculated with six different ROI fields for each sample (*n *= 3) using ImageJ software (NIH, Bethesda, MD, USA).

SEM was employed when cells had attached on different samples for 2 days. Briefly, at the time point, the samples were transferred to new 24-well culture plates, rinsed with PBS gently to remove loosely attached cells three times and then fixed with 2.5% glutaraldehyde for 30 min. Following three rinses with water, the fixed samples were dehydrated through a series of graded alcohol solutions (50%, 60%, 70%, 80%, 90% and 100%) and air-dried overnight. Before the detection, samples were sputtered with a thin layer of platinum (Pt) using a common sputtering instrument to improve the surface conductivity.

### 2.8. ALP activity

The intracellular alkaline phosphatase (ALP) activity was evaluated quantitatively using commercially available kits. Briefly, the cells were lysed in 0.5 ml of distilled water for four cycles after osteogenic induction for 7 and 14 days. The ALP activity in lysate was evaluated colorimetrically with the Alkaline Phosphatase Assay Kit (Beyotime). It can make the colourless p-nitrophenyl phosphate convert to coloured p-nitrophenol after co-incubation for 20 min at 37°C. The results were expressed in nanomoles of produced p-nitrophenol per min (nmol min^−1^).

### 2.9. Cell differentiation assays

For the osteogenic differentiation of the cells cultured on the titanium substrates, the expression levels of ALP, osteocalcin (OCN) and runt-related transcription factor-2 (RUNX2) were determined. Briefly, the hBMSCs were seeded on the samples and cultured using osteogenic medium for 14 days. Briefly, after cell adherence, the cells were cultured using the osteogenic medium (basal medium for hBMSCs supplemented with 50 mg l^−1^ ascorbic acid and 10 mM β-glycerol phosphate). The medium was changed every 2 days. Next, total RNA was extracted from the cultured cells on the samples using the TRIzol reagent (Invitrogen Life Technologies) at the time point and subsequently converted into cDNA using a HiFi-MMLV cDNA Kit (Bioman Biotechnology Co., Ltd). The RT-PCR reactions were performed using an SYBR FAST qPCR Kit (KAPA Biosystems, Wilmington, MA, USA) on the StepOne Plus RT-PCR instrument (ABI, Carlsbad, CA, USA). GAPDH was used as a housekeeping gene. The primer sequences for each gene are listed as follows: ALP (F:5′-TCCTGTTGACACCCCAAACC-3′; R:5′-GGAAACGCAGGATTTCCCAC-3′), OCN (F:5′-CTCACACTCCTCGCCCTATTG-3′; R:5′-CGCCTGGGTCTCTTCACTAC-3′), RUNX2(F:5′-CATGTCCCTCGGTATGTCCG-3′; R:5′-ACTCTGGCTTTGGGAAGAGC-3′), GAPDH(F:5′-GTCAAGGCTGAGAACGGGAA-3′; R:5′-AAATGAGCCCCAGCCTTCTC-3′)

The osteogenic expression levels were determined based on the ΔCT, ΔΔCT and 2-ΔΔCT methods.

### 2.10. ARS staining of titanium substrates

The status of a fraction of cells grown on the surface of the titanium substrates was visualized with alizarin red S (ARS) staining. After removing the cell-cultured scaffolds at 14 days, the cells on the surface were washed with PBS. After co-culturing for 14 days, the cells were fixed in 4% formaldehyde for 20 min. For the ARS staining, the cells were stained with ARS (pH 4.2) for 10 min and then visualized with a light microscope after rinsing twice with DI water.

### 2.11. Statistical analysis

Statistical analysis was conducted by SPSS Statistics 23.0 (SPSS, Inc., Al Monk, NY, USA). The experimental results were presented as the average ± standard deviation (s.d.). The differences among the three groups were investigated by one-way analysis of variance (ANOVA) and Tukey's test. The difference was statistically significant with *p* < 0.05 (*) and *p* < 0.01(**).

## 3. Results

### 3.1. Characterization

#### 3.1.1. Synthesis of Fe_3_O_4_ nanoparticles

**Figure 1.**
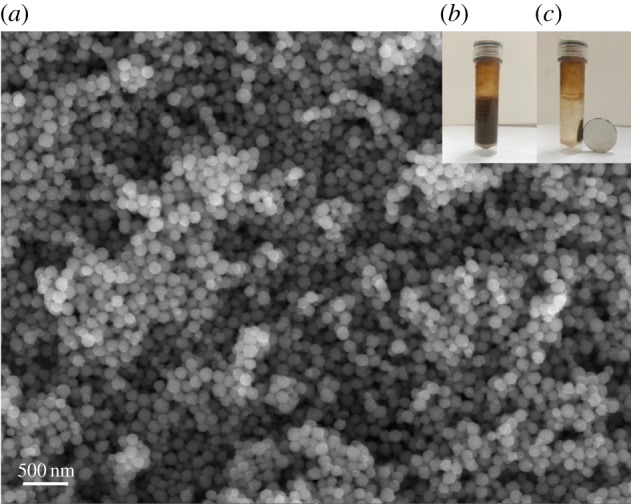
SEM image of Fe_3_O_4_ nanoparticles (*a*); Fe_3_O_4_ nanoparticles exhibited excellent colloidal stability in water (*b*); Fe_3_O_4_ nanoparticles are superparamagnetic at room temperature (*c*).

The uniform Fe_3_O_4_ nanoparticles were prepared by a modified hydro-thermal method reported by Gao *et al.* [26]. We chose the PSSMA (1: 1) (4-styrenesulfonic acid: maleic acid = 1 : 1) instead of PSSMA (3 : 1) to synthesize the Fe_3_O_4_ nanoparticles. The SEM image shown in figure 1*a* demonstrates that nearly spherical nanocrystal clusters with sizes ranging from 95 to 116 nm (average 102 nm) were formed. With bonded PSSMA molecules, Fe_3_O_4_ nanoparticles exhibited excellent colloidal stability in water (figure 1*b*). Moreover, these Fe_3_O_4_ nanoparticles are superparamagnetic at room temperature due to their small sizes with a single-crystalline structure (average 102 nm) (figure 1*c*), which was similar to the previous report by Gao *et al.* [26].

#### 3.1.2. Surface chemical composition

**Figure 2.**
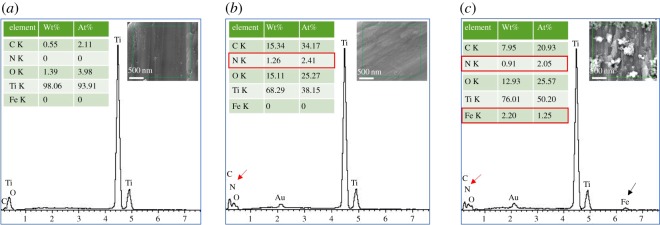
EDS data for (*a*) pTi, (*b*) PDA@pTi, (*c*) Fe_3_O_4_/PDA@pTi. The presence of nitrogen, iron elements in the EDS indicated that PDA and Fe_3_O_4_ nanoparticles were successfully immobilized on surfaces of titanium substrates.

The EDS data analysis showed that PDA (figure 2*b*) and Fe_3_O_4_ nanoparticles (figure 2*c*) are successfully immobilized on the surfaces of pTi through a one-pot surface immobilization strategy. Before the PDA coating, the titanium substrate contained titanium, carbon and oxygen elements, which indicated that a thin layer of titanium oxide covers the surface (figure 2*a*). After PDA coating, nitrogen elements appeared on the substrate, and more carbon elements can be observed than pTi (figure 2*b*). The titanium coated with PDA/Fe_3_O_4_ nanoparticles revealed iron elements on the surface (figure 2*c*). Meanwhile, the amount of oxygen increased dramatically compared to the pure PDA coating, indicating that PDA and Fe_3_O_4_ nanoparticles are successfully immobilized on the surface of pTi.

**Figure 3.**
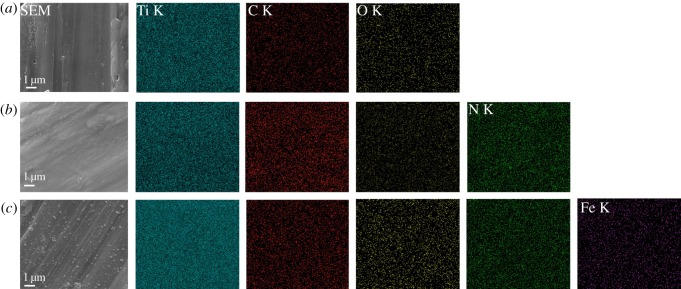
EDS elemental mapping of the area enclosed by a square in SEM image showing the distribution of Ti, C, O, N and Fe elements over the different surfaces of titanium substrates ((*a*) pTi, (*b*) PDA@pTi, (*c*) Fe_3_O_4_/PDA@pTi).

The distributions of the chemical elements on the surface of the samples observed by EDS elemental mapping are shown in figure 3. The images (figure 3*c*) indicate that Ti, C, N, O and particularly Fe are uniformly distributed on the titanium substrates.

#### 3.1.3. Surface morphology and water contact angle

**Figure 4.**
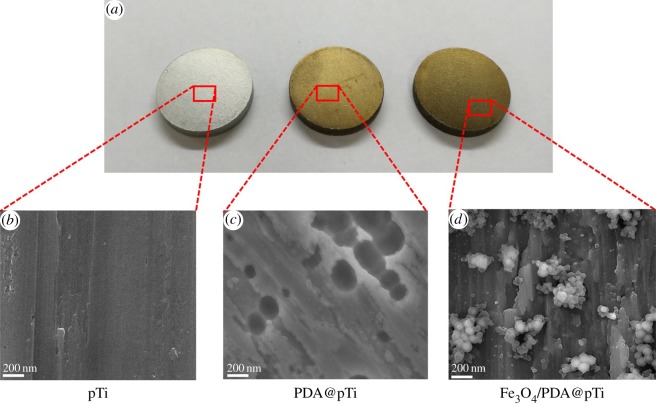
Structural properties of immobilization on surfaces: (*a*) macroscopic view of pTi, PDA@pTi and Fe_3_O_4_/PDA@pTi; (*b*) SEM image of pTi surface; (*c*) SEM image of PDA@pTi surface and (*d*) SEM image of Fe_3_O_4_/PDA@pTi surface.

SEM was used to further observe the surface morphology of the samples. As shown in figure 4*c*, the change in surface morphology was not obvious after PDA coating, while there is a dramatic change in surface morphology after coating with Fe_3_O_4_ nanoparticles. Small agglomerates with a ball-like structure, a typical form for Fe_3_O_4_ nanoparticles, appeared on the titanium surface coated by PDA/Fe_3_O_4_ nanoparticles (figure 4*d*), indicating the immobilization of Fe_3_O_4_ nanoparticles.

**Figure 5.**
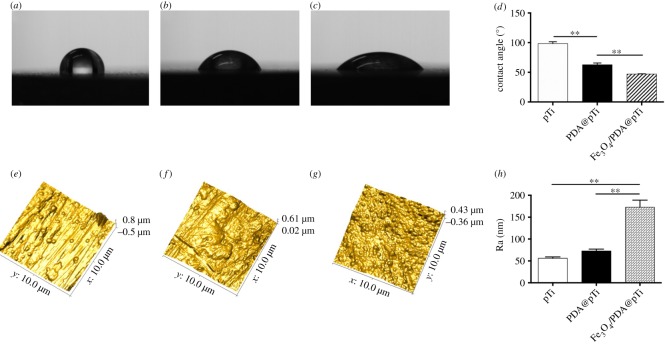
Water contact angle measurement for the (*a*) pTi, (*b*) PDA@pTi, (*c*) Fe_3_O_4_/PDA@pTi. (*d*) Average water contact angle analysis. Surface AFM images of the (*e*) pTi, (*f*) PDA@pTi, (*g*) Fe_3_O_4_/PDA@pTi. (*h*) Surface roughness analysis. For each group, *n* = 3; *p < 0.05, **p < 0.01.

It was found that the PDA coating slightly increased the surface roughness of the titanium surface (figure 5*f,h*), and the Fe_3_O_4_ nanoparticle coating significantly increased the surface roughness (figure 5*g*,*h*). Moreover, the water contact angles for the PDA coating samples significantly decreased compared with the bare titanium samples (figure 5*b*,*d*), and the Fe_3_O_4_ nanoparticle coating further substantially decreased (figure 5*c*,*d*), indicating a significant increase in wettability from the PDA and Fe_3_O_4_ nanoparticle coating on the titanium samples.

#### 3.1.4. Magnetic properties

**Figure 6.**
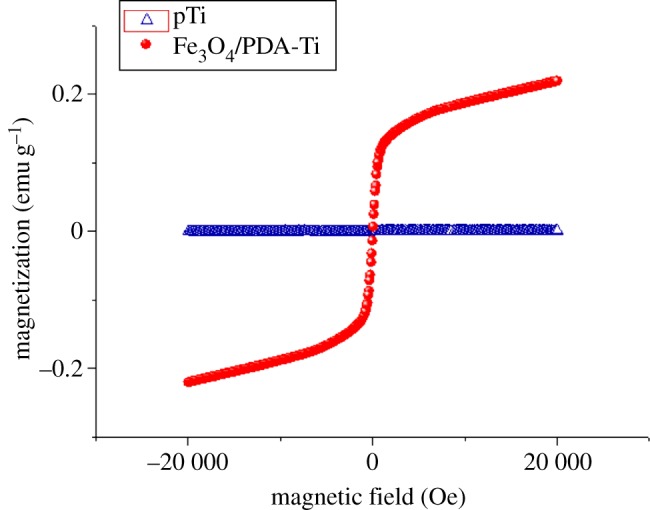
Magnetic properties of the titanium substrates analysed by a magnetization hysteresis curve. The sphere (red) represents Fe_3_O_4_/PDA@pTi; the triangle (blue) represents pTi.

The VSM was applied to measure the magnetic properties of the PDA-assisted Fe_3_O_4_ nanoparticle-coated samples, and the magnetic hysteresis (M–H) curves are depicted in figure 6. The Fe_3_O_4_ nanoparticle-coated samples exhibited a saturation magnetization of 0.22 emu g^−1^, indicating the Fe_3_O_4_ nanoparticles have been successfully incorporated on the titanium surface.

#### 3.1.5. Stability of the Fe_3_O_4_ nanoparticle coating

**Figure 7.**
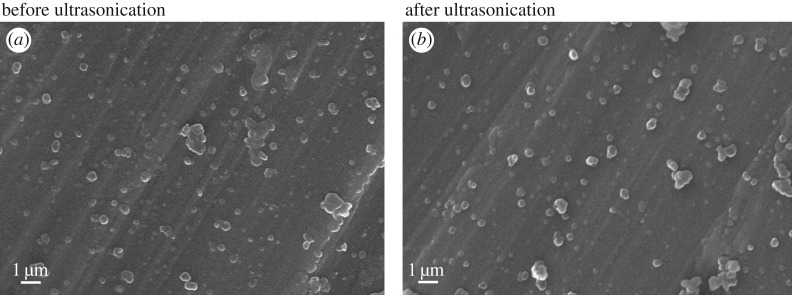
Adhesion stability of the PDA layer and Fe_3_O_4_ nanoparticles on surfaces of titanium substrates. PDA-assisted titanum substrates were investigated by SEM before (*a*) and after (*b*) exposure to ultrasonication in a water bath for 1 h.

Ultrasonication was used to evaluate the stability between the titanium substrate and Fe_3_O_4_ nanoparticle coating. As shown in figure 7, 90 ± 3.42% of Fe_3_O_4_ nanoparticles remained on the substrate surfaces after ultrasonication for an hour, and the PDA coating was also retained on the substrates, which suggested that there was strong adhesion between the titanium substrates and Fe_3_O_4_ nanoparticle coating.

### 3.2. Biological properties

#### 3.2.1. Cytotoxicity and cell proliferation

**Figure 8.**
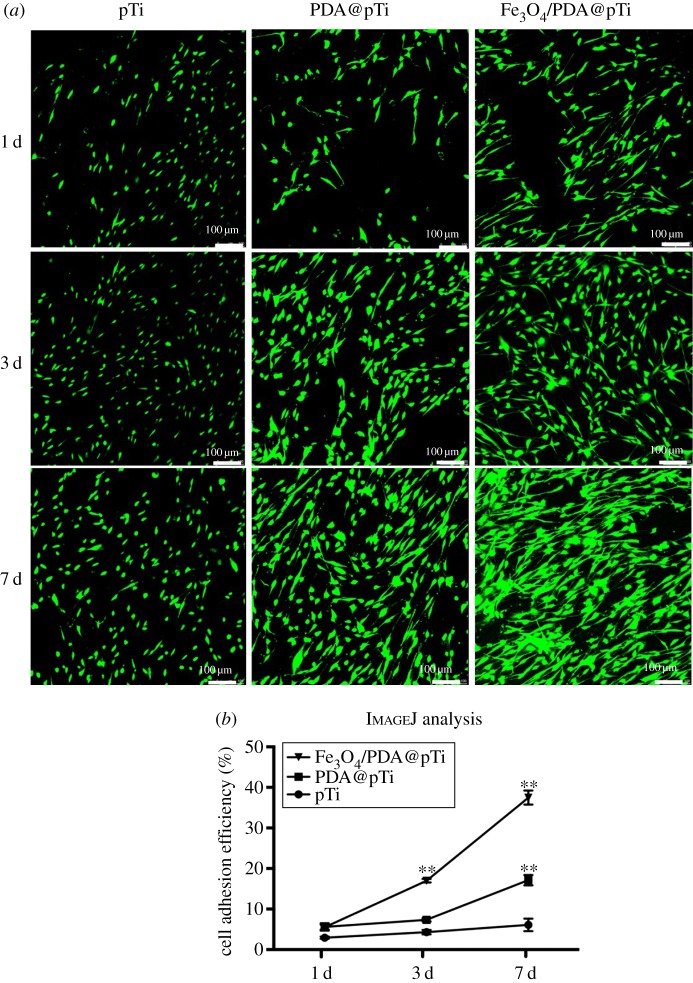
(*a*) Fluorescence images of LIVE/DEAD staining of BMSCs cells after culturing for 1, 3 and 7 days on the surfaces of Fe_3_O_4_/PDA@pTi, PDA@pTi and pTi. (*b*) ImageJ analysis of cell adhesion efficiency. For each group, *n* = 6; *p < 0.05, **p < 0.01.

The LIVE/DEAD^®^ viability/cytotoxicity kit was used to qualitatively assay the cytotoxicity of the different titanium substrates after culturing for 1, 3 and 7 days, and the results are shown in figure 8. A larger number of live and spindle cells with obvious cell–cell junctions and formed network links can be observed from the PDA@pTi and Fe_3_O_4_/PDA@pTi samples. Nevertheless, a small number of round cells with few intercellular connections can be noticed on the surface of the pTi substrates. These results indicate that the PDA and Fe_3_O_4_ nanoparticle coating can not only improve the cell proliferation and attachment on the coated Ti surface but also stimulate cell–cell interactions.

**Figure 9.**
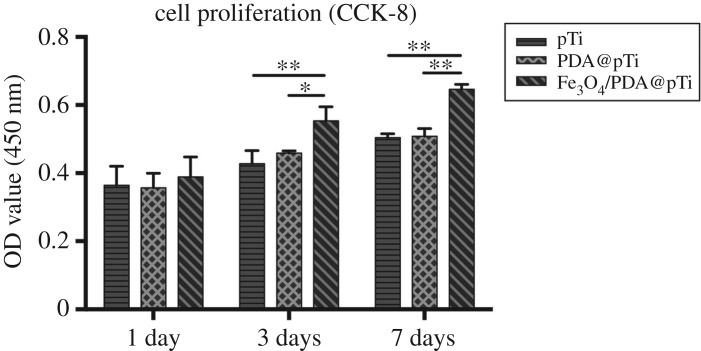
Measurement of BMSCs cells proliferation by CCK-8 assay after 1, 3 and 7 days incubation. For each group, *n* = 3; *p < 0.05, **p < 0.01.

The cell proliferation was investigated by the CCK-8 assay after being cultured for 1, 3 and 7 days, and the number of cells on the samples increased continuously up to 7 days. We found that the group of Fe_3_O_4_/PDA@pTi showed significantly greater cell proliferation than the group of pTi and PDA@pTi (figure 9). Additionally, it was found that there were no significant differences in cell proliferation between the pTi and PDA@pTi groups. Therefore, these results indicated that the Fe_3_O_4_ nanoparticle coating promoted hBMSCs proliferation on the titanium substrates.

#### 3.2.2. Cell attachment

**Figure 10.**
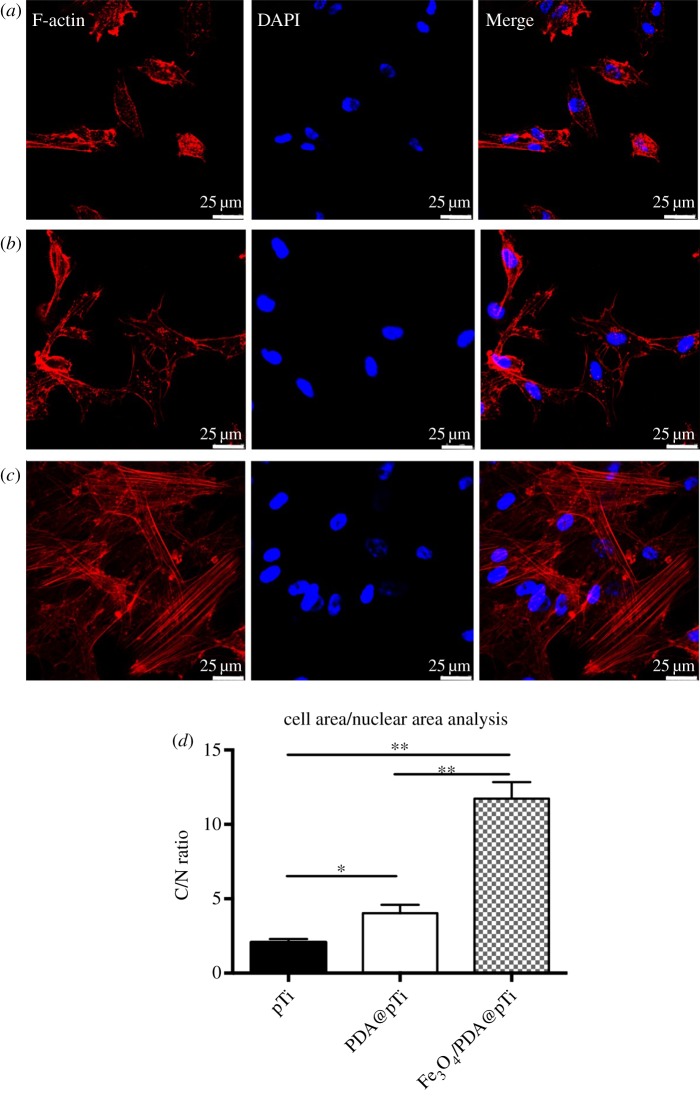
Fluorescent staining of BMSCs cells adhered on (*a*) pTi, (*b*) PDA@pTi, (*c*) Fe_3_O_4_/PDA@pTi after culturing for 1 day. BMSCs cells morphology analysis (*d*). For each group, *n* = 6; *p < 0.05, **p < 0.01.

The cell adhesion behaviour was investigated by fluorescence staining for F-actin and nuclei with rhodamine-phalloidin and DAPI, respectively. As shown in figure 10, the fluorescence intensity and quantity of F-actin on the group of Fe_3_O_4_/PDA@pTi were the highest compared with those on pTi and PDA@pTi groups. On the other hand, cells in the PDA@pTi groups were distributed better than those on the pTi groups. To quantitatively analyse the cell adhesion behaviour, we calculated the C : N ratio (cell overall area : nucleus area) using ImageJ software. The statistical results in figure 10*d* showed that the C : N ratios on the groups of Fe_3_O_4_/PDA@pTi were higher when compared with pTi and PDA@pTi groups, and the groups of PDA@pTi were also higher than pTi groups. Thus, these results indicated that the Fe_3_O_4_/PDA and pure PDA coating both promote cell adhesion. Moreover, the higher surface roughness of Fe_3_O_4_/PDA@pTi may contribute to cell attachment.

#### 3.2.3. Cell morphology

**Figure 11.**
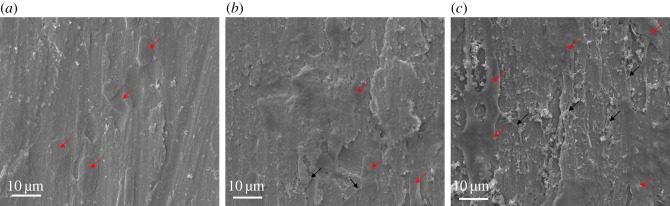
SEM images of BMSCs cells cultured on (*a*) pTi, (*b*) PDA@pTi, (*c*) Fe_3_O_4_/PDA@pTi for 2 days. Red arrows indicate cells grown on the surfaces of the titanium substrates, and black arrows indicate lamellipodia extensions.

The morphology of the cells cultured on titanium substrates was estimated by a scanning electron microscope (SEM). The mitosis of hBMSCs was in activity, and the number of cells increased over time. As shown in figure 11, after 2 days of culture, there were plentiful cells found in the Fe_3_O_4_/PDA@pTi group. Moreover, the quantity of lamellipodia extensions between the adjacent cells on the surface of the Fe_3_O_4_/PDA@pTi groups is significantly higher than the other two groups, especially in the bare pTi groups, which was in line with the proliferation data of the CCK-8 assays and fluorescent staining assays.

#### 3.2.4. ALP activity

**Figure 12.**
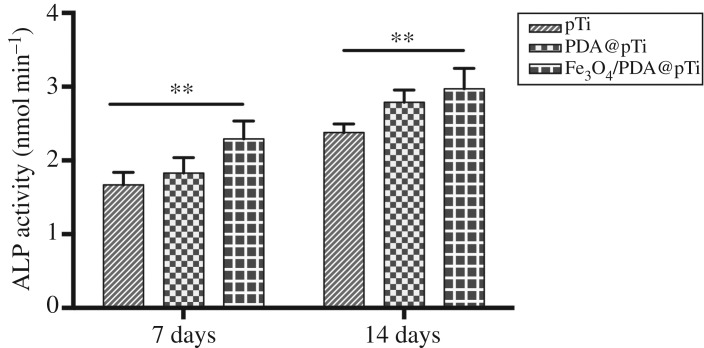
ALP activity of BMCSs cells cultured on the pTi, PDA@pTi and Fe_3_O_4_/PDA@pTi arrays after osteogenic induction for 7 and 14 days. For each group, *n* = 3; *p < 0.05, **p < 0.01.

The ALP activity levels after 7 and 14 days of incubation were examined to evaluate the bone differentiation activity of surface-modified titanium substrates. The results in figure 12 show that ALP activity for all three groups gradually increased for up to 14 days, and the ALP activity in the Fe_3_O_4_/PDA@pTi groups was much higher than that in the other two groups. The groups of PDA@pTi were slightly higher than pTi groups, but no significant differences were observed, which implied that the magnetic properties of Fe_3_O_4_ nanoparticles significantly increased intracellular ALP synthesis and secretion *in vitro*.

#### 3.2.5. Cell differentiation

**Figure 13.**
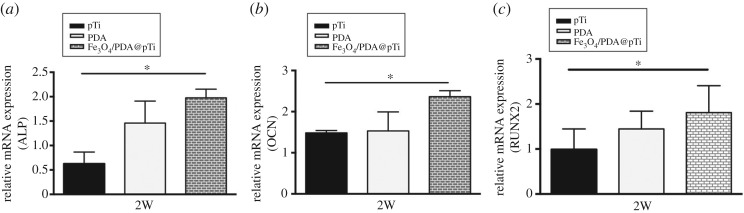
Relative mRNA expression of ALP (*a*), OCN (*b*) and RUNX2 (*c*). For each group, *n* = 3; *p < 0.05, **p < 0.01.

An important feature of Fe_3_O_4_/PDA@pTi is its magnetic properties, which could accelerate cell osteogenic differentiation. To study the role of osteogenesis by magnetic properties in Fe_3_O_4_/PDA@pTi, the mRNA expression levels of ALP, OCN and RUNX2, which reflect the grade of osteogenic differentiation of hBMSCs, were determined by quantitative RT-PCR tests at 14 days. As shown in figure 13, compared with the unmodified pTi groups, the Fe_3_O_4_/PDA@pTi groups showed significantly higher gene expression of ALP, OCN and RUNX2 in hBMSCs after 14 days of osteogenic induction culture (*p* < 0.05). However, the difference for ALP, OCN and RUNX2 gene expressions between the PDA@pTi and pTi groups has no statistical significance. These results implied that the Fe_3_O_4_ nanoparticles on the surface of the titanium substrates promote the osteogenic differentiation of hBMSCs.

#### 3.2.6. ARS staining of titanium substrates

**Figure 14.**
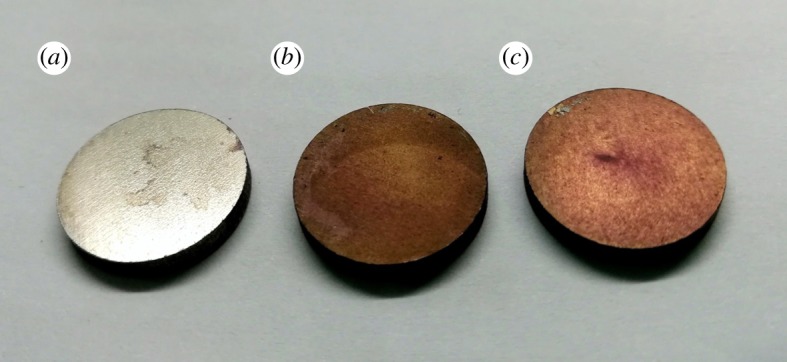
ARS staining of fixed cells on the surface of (*a*) pTi, (*b*) PDA@pTi and (*c*) Fe_3_O_4_/PDA@pTi.

The status of a fraction of cells grown on the surface was visualized by ARS staining. ARS assays displayed much redder staining images of cells on the surface of Fe_3_O_4_/PDA@pTi groups (figure 14*c*).

## 4. Discussion

Owing to good load-bearing properties, outstanding biocompatibility and superior corrosion resistances, titanium has been applied for bone defect repair [1]. However, further improving the osteoconductive and osteoinductive properties of the titanium substrates is still a hot topic for clinical applications [4–8,28,29]. Magnetic properties are considered an effective stimulator of cell proliferation, migration and differentiation and could be used to repair and heal damaged tissues [9–15,30]. In this work, we used magnetic properties aiming to stimulate the osteogenic differentiation of hBMSCs for bone regeneration. Previous studies have already proclaimed that the magnetic scaffolds incorporating MNPs promote the proliferation and osteogenic differentiation of cells, including hBMSCs [10,12–16,30]. Another previous study revealed that Fe_3_O_4_ nanoparticles synthesized with different amounts of PSSMA (poly 4-styrene sulfonic acid-co-maleic acid) are superparamagnetic at room temperature [26]. The previous investigations have already determined that Fe_3_O_4_ nanoparticles with an adequate biocompatible coating do not have cytotoxic effects on cell development either *in vitro* or *in vivo* [31]. Therefore, we hypothesized that if we apply magnetic Fe_3_O_4_ nanoparticles to modify the surface of titanium substrates as a surface immobilization strategy, we might generate the intended effects for bone regeneration. To test this hypothesis, we should first find an appropriate surface immobilization strategy.

Previously, a facile and simple surface immobilization method based on PDA was revealed [22]. The PDA immobilization methods are inspired by the amino acid composition of mussel adhesive proteins. The PDA coating was demonstrated to spontaneously deposit thin films on a wide array of material surfaces and allow for the formation of multifunctional organic/inorganic ad-layers [24]. Kang and co-workers reported a one-step multipurpose surface functionalization by the one-step immersion of substrates in a one-pot mixture of a specific target and a PDA agent, which is a simpler strategy for surface modification that can be used for the surface immobilization of various materials [25]. In this study, we chose the one-step PDA-assisted coating as a proper surface immobilization strategy to improve the osteoconductive and osteoinductive properties of titanium substrates. Our results showed that the PDA-assisted immobilization efficiency of Fe_3_O_4_ nanoparticles was up to 90 ± 3.42% through ultrasonication for an hour. Thus, we confirmed that the PDA-assisted coating effectively immobilized Fe_3_O_4_ nanoparticles on the surface of titanium substrates and obtained a high immobilization efficiency.

Owing to the superparamagnetic nature of Fe_3_O_4_ nanoparticles coated on the surface of titanium substrates, the Fe_3_O_4_/PDA@pTi exhibited certain magnetic properties with a saturation magnetization of 0.22 emu g^−1^ (the weight of the titanium is much larger than that of the Fe_3_O_4_ nanoparticles). This value was significantly lower than that reported elsewhere for nanocomposite scaffolds incorporated with MNPs (4.8 emu g^−1^) [15] but higher than the paramagnetic nanofibrous composite film incorporated with γ-Fe_2_O_3_(0.049 emu g^-1^) [10,13]. The coated substrate has exhibited excellent osteogenesis activity despite the low level of saturation magnetization. Above all, we developed a PDA-assisted Fe_3_O_4_ nanoparticle surface modification strategy relying on tight and integrated magnetic properties, ultimately improving the osteogenesis of the titanium substrates.

Cell adhesion is the first essential step when cells are in contact with an adequate surface in bone regeneration, which will greatly affect the morphology and ability of cell proliferation and differentiation [32]. Therefore, cell adhesion and subsequent growth are important indicators of whether a material can be used as a substitute for bone regeneration. In this work, hBMSCs were cultured on the titanium substrates to explore the state of cell growth. Compared to that on pTi, the fluorescence intensity and quantity of F-actin was higher on PDA@pTi, which is mainly due to the viscosity of PDA that facilitates the cell attachment. The result of Fe_3_O_4_/PDA@pTi was better than PDA@pTi, which is mainly due to the tiny magnetic field provided by the MNPs that facilitate the cell attachment and further proliferation. After 7 days of cell culture, both LIVE/DEAD and CCK-8 assays showed that the cell adhesion and proliferation showed an increasing trend with the increase of inoculation time and loading of Fe_3_O_4_ nanoparticles, suggesting that all substrates in this experiment have good biocompatibility and that Fe_3_O_4_ nanoparticles coated onto titanium substrates can greatly improve the proliferation and growth of hBMSCs.

In addition to cell adhesion and proliferation, cell differentiation is crucial for bone regeneration. ALP is an enzyme that is an early differentiation marker of hBMSCs and produced by the cells during osteogenic differentiation [33]. It was found that the levels of ALP activity were higher in the Fe_3_O_4_/PDA@pTi groups compared with those in the pTi and PDA@pTi groups at both 7 and 14 days. Osteogenic differentiation of hBMSCs was followed by evaluating the mRNA expression levels of ALP, OCN and RUNX2 by quantitative RT-PCR tests at 14 days. It was found that the levels of ALP, OCN and RUNX2 in Fe_3_O_4_/PDA@pTi at 14 days were significantly higher than in the other two groups. Thus, these results indicated that the Fe_3_O_4_ nanoparticle coating plays a major role in osteogenic differentiation.

Similar results have been obtained in previous studies on the osteoinduction of MNPs. Bock *et al.* [34] indicated the addition of MNPs in hydroxyapatite and collagen scaffolds using a dip-coating technique that supports significantly higher adhesion and proliferation of human bone marrow stem cells *in vitro*. Singh *et al.* [14] demonstrated that the addition of MNPs to biopolymer scaffolds increased the adhesion and differentiation of osteoblast cells *in vitro* and the bone formation *in vivo* without applying external magnetic fields. One of the mechanisms is probably that each magnetic nanoparticle on the titanium substrate could be regarded as a single magnetic nanofield to provide micromotion at the interface between the cells and titanium substrates. The tiny magnetic fields might affect the ion channels on the cell membrane and trigger the mechanotransduction pathway, leading to increased cell growth, proliferation and differentiation [35]. Another mechanism is possible that MNPs on the titanium substrates attract and take up the growth factor or other bioagents via a driving magnetic force [34]. Recently, Zhu *et al.* [36] demonstrated that MNPs altered the composition of protein coronas and ultimately contributed to the increased concentration of proteins related to calcium ions, G-protein coupled receptors and MAPK/ERK cascades, resulting in promoting MC3T3-E1 cell proliferation.

It is known that surface wettability and roughness are two important factors for cellular response to the substrates [37,38]. The significant decrease in the water contact angle caused by the PDA coating on the titanium substrates and further by the PDA-assisted Fe_3_O_4_ nanoparticle coating indicated that the surface wettability was substantially enhanced. The surface morphology observed using AFM demonstrated that the surface roughness increased after PDA coating and was further dramatically increased by the PDA-assisted Fe_3_O_4_ nanoparticle coating. Costa *et al.* [38] demonstrated that surface morphology and roughness improve cell adhesion and migration in osteoblast cells.

At this point, the cellular reactions promoted by the magnetic titanium substrates need to be understood. First, we considered that the improved wettability and roughness of magnetic titanium substrates would be acknowledged in certain respects. However, in addition to the altered wettability and roughness, other properties of the magnetic titanium substrates, such as magnetic properties, may be additional reasons for the cellular reactions. Herein, we speculated the possibility of a synergistic role of the wettability and roughness with magnetic properties of Fe_3_O_4_ nanoparticles, resulting in promoting the proliferation and differentiation of hBMSCs.

Thus, we herein demonstrate that PDA-assisted Fe_3_O_4_ nanoparticle coating is a promising method to modify the surface of titanium substrates to promote cell growth, proliferation and differentiation of hBMSCs *in vitro*. In future work, the application of titanium substrates with PDA-assisted Fe_3_O_4_ nanoparticle coatings for bone defect repair will be further investigated.

## 5. Conclusion

It was demonstrated that the PDA-assisted Fe_3_O_4_ nanoparticle coating surface immobilization strategy could endow the modified titanium substrates with significant osteogenic activity. This new coating efficiently promoted cell attachment, proliferation and differentiation of hBMSCs *in vitro*. These findings indicated that PDA-assisted surface immobilization is a simple, effective method to tightly immobilize osteoconductive and osteoinductive components onto biomaterial surfaces. Further investigation should be carried out to use PDA-assisted Fe_3_O_4_ nanoparticle coatings to modify other biomaterials for the treatment of bone defects.

## Data accessibility

All primary data collected for this study have been deposited with Dryad and can be accessed at (http://dx.doi.org/10.5061/dryad.ms750) [39].
